# Estimating the Impact of Nanophases on the Production of Green Cement with High Performance Properties

**DOI:** 10.3390/ma13184197

**Published:** 2020-09-21

**Authors:** Inas A. Ahmed, Najlaa S. Al-Radadi

**Affiliations:** 1 Department of Chemistry, Faculty of Science, King Khalid University, Abha 62224, Saudi Arabia; 2 Department of Chemistry, Faculty of Science, Taibah University, Al-Madinah Al-Munawarah 14177, Saudi Arabia; nsa@taibahu.edu.sa

**Keywords:** nanophases, β-C_2_S, calcium sulfoaluminate, monosulfate, hydration properties, compressive strength

## Abstract

Ordinary Portland cement (OPC) production is energy-intensive and significantly contributes to greenhouse gas emissions. One method to reduce the environmental impact of concrete production is the use of an alternative binder, calcium sulfoaluminate cement, which offers lower CO_2_ emissions and reduces energy consumption for cement production. This article describes the effect of adding nanophases, namely belite, calcium sulfoaluminate, calcium aluminum monosulfate (β-C_2_S, C_4_A_3_S, and C_4_AS, respectively) on OPC’s properties. These phases are made from nanosubstances such as nano-SiO_2_, calcium nitrate (Ca(NO_3_)_2_), and nano-aluminum hydroxide Al(OH)_3_ with gypsum (CaSO_4_·2H_2_O). The impact of β-C_2_S, C_4_A_3_S, and C_4_AS nanophases on the capabilities of cements was assessed by batch experimentations and IR, XRD, and DSC techniques. The results showed that the substituting of OPC by nano phases (either 10% C_4_A_3_S or 10% C_4_A_3_S and 10% β-C_2_S) reduced setting times, reduced the water/cement ratio and the free-lime contents, and increased the combined water contents as well as compressive strength of the cement pastes. The blends had high early and late compressive strength. The IR, XRD, and DSC analyses of the blends of 10% C_4_A_3_S or 10% C_4_A_3_S and 10% β-C_2_S cement displayed an increase in the hydrate products and the presence of monosulfate hydrate. The addition of 10% C_4_AS or 10% C_4_AS and 10% β-C_2_S to OPC reduced the setting times, decreased the W/C ratio, free lime, the bulk density, and increased the chemically-combined water and compressive strength. Overall, the results confirmed that the inclusion of the nanophases greatly enhanced the mechanical and durability properties of the OPCs.

## 1. Introduction

Nanoparticles (NPs) display distinctive physicochemical properties that may help in the development of more effective resources than those currently existing. Extremely fine-sized NPs give favorable features due to their high surface area [1]. Applying nanomaterials to cement and concrete production could lead to improvements in civil infrastructure. The reduction in particle size could lead to the much faster setting and solidifying of cement pastes due to strong electrostatic attraction forces and high surface areas. Scientists have grasped that NPs that are consistently spread in a cement paste will quicken cement hydration due to their high activity [2]. Additionally, NPs fill pores and increase strength, which enhances the micro-structure of cement pastes and the borders between cement pastes and aggregates in concrete [3].

CSA (sulfoaluminate cement) resources have been observed to be very attractive, high-performance materials. Their use allows for rapid strength gain and endurance to various hostile surroundings, which makes them commonly used in civil engineering. They are chemically compatible with and adhere strongly to ordinary Portland cement (OPC), including carbonated OPC. CSA self-dries more readily than OPC compositions, and this self-drying provides protection against the corrosion of embedded steel, even in cyclical exposure to chloride-containing seawater. The high sulfate content in CSA improves its durability to seawater attacks, and it not only saves power and limestone resources but also diminishes CO_2_ emissions [4]. Though sulfoaluminate cement has several flaws, namely its low content and slow hydration of C_2_S, these cause no considerable increase in its later strength [5]. As an innovative kind of CSA, high β-C_2_S-sulfoaluminate cement (HBSAC) has an optimized mineral component attained though C_4_A_3_S reduction and C_2_S addition [6], which effectively assures the growth of the late cement’s strength and reduces the quantity of pure gypsum and Al_2_O_3_ supplies. HBSAC might diminish impacts on the environment and global warming produced by the production of OPC [7]. The lower expected amount of carbonates and the reduction of CO_2_ emissions, related to the lower firing temperature compared to OPC clinkers, are significant benefits of those formulations [8].

Martin-Sedeno et al. [9] examined the hydrating procedures of the three Al-rich belite sulfoaluminate cements (BSAC). Živica [10] assessed mixtures of BSAC containing diverse pozzolans (blast-furnace slag, fly-ash, and silica-fume) to investigate setting time and developing compressive strength. Mortar set using ettringite-rich sulfoaluminate cements showed quicker hydrating and high self-drying behaviors than one containing OPC [11]. A mortar made using mixtures of BSAC and ordinary cement displayed a more advanced defense against rusting of steel than a blast furnace slag and ordinary cement binder [12]. Additionally, a concrete-mixture manufactured with BSAC showed an initial growth of strength and outstanding sulfate resistance, though it also showed poor working ability and greater levels of carbonation compared to an ordinary cement formulation [13]. CaAl_2_O_4_, C_3_A, and iron/gehlenite are the minor phases of calcium sulfoaluminate cement, and its chief raw materials are yeelimite, β-C_2_S, gypsum [14,15,16], ettringite (AFt), and monosulfate (AFm), along with a calcium silicate hydrate, Al_2_O_3_ and Fe_2_O_3_ [17].

This research aimed to explain the effect of adding more nanophases during green cement production along with high-performance assets by means of few waste products. The effect of nanophases such as β-C_2_S (BC), CSA, calcium sulfoaluminate-β-C_2_S (CSAB), calcium aluminum monosulfate (CMS), and calcium aluminum monosulfate-β-C_2_S (CMSB) mixes on the properties of cement pastes was examined. The effect of replacing cements using nanophases on the function of cement cured at 3, 7, 28, and 90 days was evaluated by analyzing water consistency, combined H_2_O contents, CaO contents, compressive strength, and bulk density. IR, XRD, and DSC methods were used in the identification of hydrating products.

## 2. Experiment

### 2.1. The Starting Resources

The used materials included ordinary Portland cement provided by Suez Cement Company, Suez, Egypt. Sodium silicate and HCL were provided from Elgomhouria Company Cairo, Egypt. Sodium silicate (Na_2_SiO_3_) was acid-hydrolyzed for nano-silica synthesis using diluted HCl, 0.5 N, which was then gradually added while stirring in Na_2_SiO_3_ at 60 °C at a pH between 1 and 2. For the acid hydrolysis of Na_2_SiO_3_, the above-mentioned process of stirring the solution was carried out for 30 min at 60 °C. The solution–gel mix was washed well to completely eliminate NaCl, then the mix was dried at 50 °C for 3 h [18,19].

Nano-Al hydroxide was synthesized from fine Al-dross (0.1 mm) that was made to precipitate well and was provided by the Misr Company for Aluminum, Nag’ a Hammady, Egypt (MCA), and sheer aluminum hydroxide (Al(OH)_3_) was later collected via leaching using a commercially available dilute HCl solution. Aluminum dross powder was leached using 1:4-ratioed hydrogen chloride/tap-water mix for about 8 h at 100 °C, yielding a deposit comprising most aluminum ions (60%) for the precipitation of a pure Al-hydroxide gel in the HCl-leaching filtrate in a 1:1commercial ammonia solution at a pH of 8 [18,20].

After adding HNO_3_ to CaCO_3_ (provided from Elgomhouria Company Cairo, Egypt) in a 1:1 ratio, the mixture was stirred well to make sure that the calcium carbonate completely dissolved. To achieve solidification, the mixture was evaporated at 50–60 °C, and then the powder was allowed to dry for 24 h and later held in a desiccator.

### 2.2. Synthesis of ß-C_2_S, C_4_A_3_S, and C_4_AS Nanophases

All these phase syntheses were done as discussed in our previous study [18]; using an appropriate molar ratio of nano silica (NS) and calcium nitrate, C_2_S was made and the dried components were stirred for about 30 min with a ceramic ball mill with 2 balls to achieve full homogeneousness; then the mixture was set at 1150 °C for 2 h, milled, and saved using sealed bottles until being used for the experiment.

Using an appropriate molar ratio of nano-Al(OH)_3_-(AH_3_), calcium nitrate, and pure CaSO_4_·2H_2_O, calcium sulfualuminate phase (CSA)was made, and the dried components were stirred using a ball mill for 30 min to achieve whole homogeneousness before being left to set at 1290 °C for 2 h and then milled and saved using sealed bottles.

The calcium aluminum monosulfate (C_4_AS) mix was mainly composed of 4 moles of Ca(NO_3_)_2_, Al(OH)_3_, and CaSO_4_·2H_2_O. After mixing and firing at 1350 °C, the fired phases were C_4_A_3_S, 2CaSO_4_, and 6CaO. These phases, after hydration, provided monosulfate hydrate. The major constituents of every stage were gauged using X-ray diffraction (XRD) and recorded on a Philips PW 1050/70 diffractometer (Philips, Amsterdam, Hollande) using a Cu–Kα source with a post sample Kα filterant, a scanning speed of 1 s/step, a range of 5 to 50 (2θ°), and a resolution of 0.05°/step).

### 2.3. Concrete Mix Proportions and Test Methods

Two series of composite cements were prepared from OPC, β-C_2_S, calcium aluminum sulfate, and monosulfate mix phases. The dry constituents were mixed for 30 min using a ceramic sphere grinder to achieve full homogeneousness. The mortars are prepared according to American Standard Test Methods (ASTM) Designation C-191 [21]. Each specimen was cast within stainless-steel moldings—0.5-inch cubes that was demolded 24 h later and treated using clean water from the faucet at 23.0 ± 2 °C until the experiment period. To ensure the same workability of the entire specimen, the water used for mixing was measured.

Corresponding to ASTM: C191 description using Vicat Apparatus; the water consistency and setting times for each mixture were decided. After the predetermination of time, the hydration of cement pastes was performed on the crushed paste cubes after the determination of compressive strength by a compressive strength machine of SEIDNER, Riedinger, W. Germany, with maximum capacity of 60 KN force. The stopping solution was made with methanol and propanone at a 1:1 volume [22]. After setting at 1000 °C for 1 h, the chemically-combined H_2_O contents were determined as the loss on ignited weight basis. The crystalline phases of pastes were detected by by X-ray diffraction (XRD) and recorded on a Philips PW 1050/70 diffractometer (Philips, Amsterdam, Hollande) using a Cu–Kα source with a post sample Kα filterant, a scanning speed of 1 s/step, a range of 5 to 50 (2θ°), and a resolution of 0.05°/step). For the verification of the procedure that used chemical- and machine-driven tests, a few certain hydrated samples were inspected for Differential Scanning Calorimetry (DSC) analysis using a LABSYS DSC 1600 rod differential thermal analyzer (SETARAM, Caluire-et-Cuire, France), and FTIR-spectroscopy using potassium bromide (KBr) was conducted with a Genesis-II FT-IR spectrometer (ALT, San Diego, CA, USA) at a wavelength of 400–4000 cm^−1^. The actual particle sizes of materials were measured by transmission electron microscopy (TEM) with the JEM-HR-2001 model (JEOL, Akishima, Japan) which was connected with an accelerating voltage of 200.

## 3. Result and Discussions

### 3.1. Characteristics of Nanophases

Chemical constitution of starting components (wt.%) and phase composition of ordinary Portland cement (OPC) are shown in Table 1. The XRD and TEM characterization of the synthesized nano-silica and aluminum hydroxide was previously reported in our earlier study [23]. The XRD patterns confirmed that the obtained nano-SiO2 was completely amorphous, while aluminum hydroxide consisted mainly of gibbsite and bayerite phases with a low degree of crystallinity. The TEM analysis revealed that the average particle sizes of nano-silica and aluminum hydroxide were approximately 13 nm and 38 nm, respectively.

**Figure 1:**
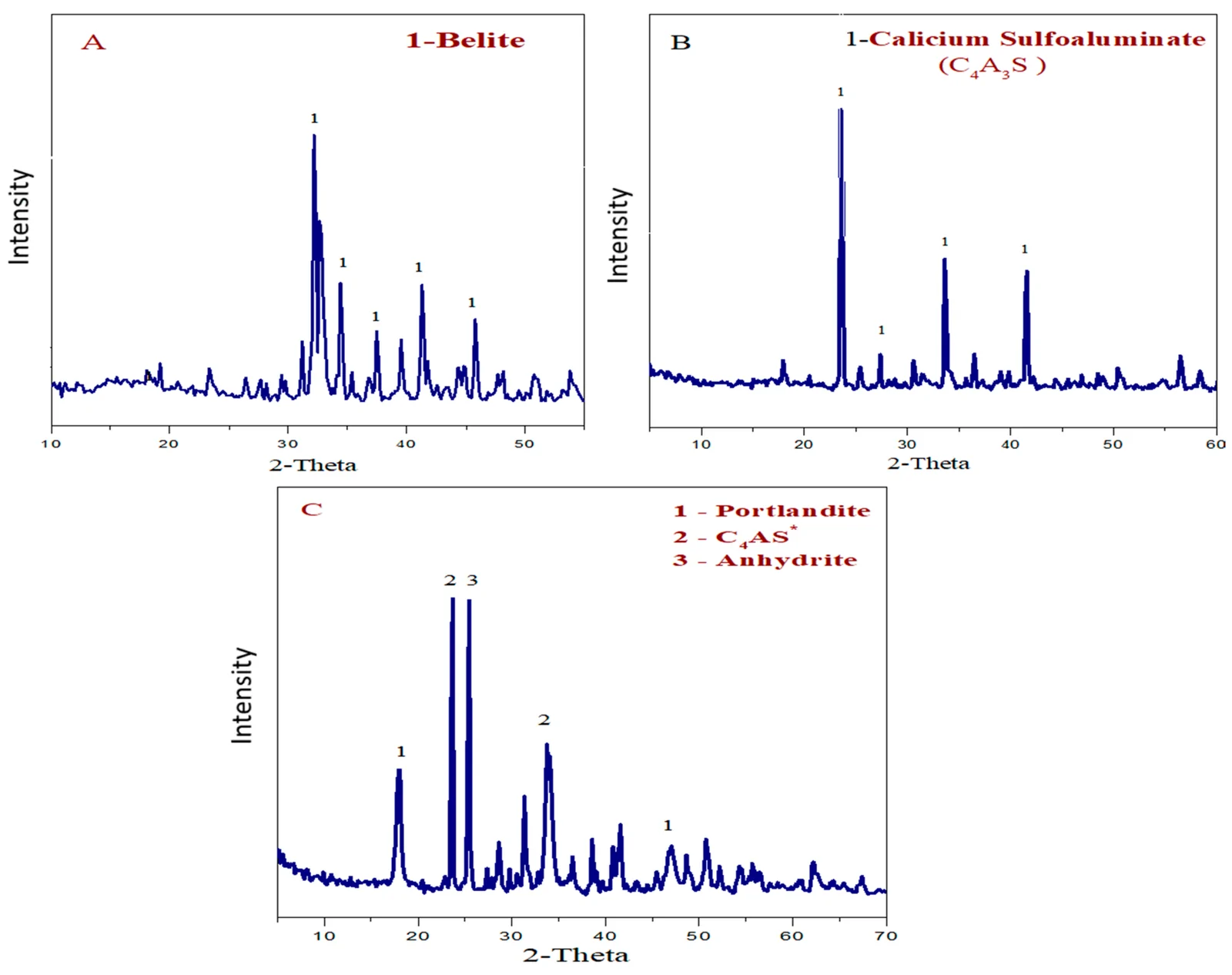
The XRD patterns of the (**A**) β-C_2_S, (**B**) calcium sulfoaluminate-β-C_2_S, and (**C**) calcium aluminum monosulfate nanophases.

The XRD-patterns of the β-C_2_S, calcium sulfoaluminate, and calcium aluminum monosulfate nanophases are shown in Figure 1. β-C_2_S’s XRD-pattern was formed by using NS and nano-Ca(NO_3_)_2_ and a temperature set at 1150 °C, as shown in Figure 1A, which displays a well-emphasized β-C_2_S peak. This can be attributed to the high specific surface of the reactants; consequently, the chemical reaction occurred at a relatively higher rate at a lower temperature. The reaction is roughly potted as [24]:

2Ca(NO_3_)_2_ → 2CaO + 4NO_2_ + O_2_ (1)

2CaO + SiO_2_ → Ca_2_SiO_4_ (2)

Corresponding to the work of Kurdowski et al. [25], the energy essential for the production of β-C_2_S was found to be about 1350 kJ/kg. The outcomes of current study revealed that the energy consumed for β-C_2_S production was diminished by 14%.

The XRD patterns of C_4_A_3_S prepared from the nanomaterials Al(OH)_3_, Ca(NO_3_)_2_, and pure gypsum at 1290 °C are shown in Figure 1B. The XRD patterns showed a higher intensity of CSA at lower temperatures than usual due to the reaction between the ultrafine nano-aluminum hydroxide, calcium nitrate, and pure CaSO_4_·2H_2_O, that had high specific surface area with high reactivity. The amplified reactivity and smaller particle size led to an increase in the rate of the reaction. The XRD patterns of the calcium aluminum monosulfate phase synthesized from 4 moles of Ca(NO_3_)_2_, Al(OH)_3_, and CaSO_4_·2H_2_O fired at 1350 °C are seen in Figure 1C. The pattern shows C_4_AS and anhydrite, as well as portlandite. These phases, after hydration, provided monosulfate hydrate. In this study, a total of six mixes in two progressions were made, as shown in Table 2.

### 3.2. Performance of OPC, CSA, CSAB, and BC Cements

#### 3.2.1. The Water Consistency

The water consistency, the initial time setting, and the final time setting of the OPC, CSA, CSAB and BC cements are shown in Table 2. The results showed that the water consistency of the CSA cement was slightly greater than that of OPC [26]. This was mainly due to the ettringite formation that required extra hydration water. The water consistency of CSAB was nearly the same as that of OPC. This was due to the fact that the hydration of calcium sulfoaluminate needed more water than OPC, whereas β-C_2_S had a lower rate of hydration than that of OPC [27,28]. Therefore, the CSAB cement had the same water consistency as OPC. The outcomes showed that the initial and final setting times shortened with the CSA and CSAB cement pastes. This was caused by the very rabid hydration of C_4_A_3_S-forming ettringite, which accounted for the fast drying of sulfoaluminate cements [15]. Additionally, by adding NPs of these phases, the average size in diameter got smaller and the setting time was reduced. As expected, the decrease in particle size tended to accelerate the cement hydration, thus causing much faster setting [29,30]. On the other hand, the addition of β-C_2_S with sulfoaluminate shortened the setting time. This may have been due to the decrease in the water demand, or it may have been due to the fine particle size of β-C_2_S that sealed gaps among the cement granules, which connected them and caused stiffening and, consequently, hardening. The initial setting time of the CSA and CSAB cement pastes required the standard specification or less than 40 min. The substitution of OPC with equal amounts of sulfoaluminate and β-C_2_S (CSAB) tended to accelerate the final setting time. This was mostly related to the development of more calcium sulfoaluminate hydrates as AFt or AFm as well as the surface area of these nano materials which accelerated the setting time.

The outcomes indicated that the water consistency of the BC cement was decreased compared to that of OPC. This was mostly related to the low hydration rate of the β-C_2_S phase. The addition of β-C_2_S to OPC shortened the setting time. Generally, however, it may be specified that the setting and solidifying of cement the result of dissolution and precipitating processes, and the particle size of the precipitate phases is a key factor in these processes. Actually, the well-known fast setting of OPCs is caused by the small size of their powder particles [29,31].

#### 3.2.2. X-ray Study

**Figure 2:**
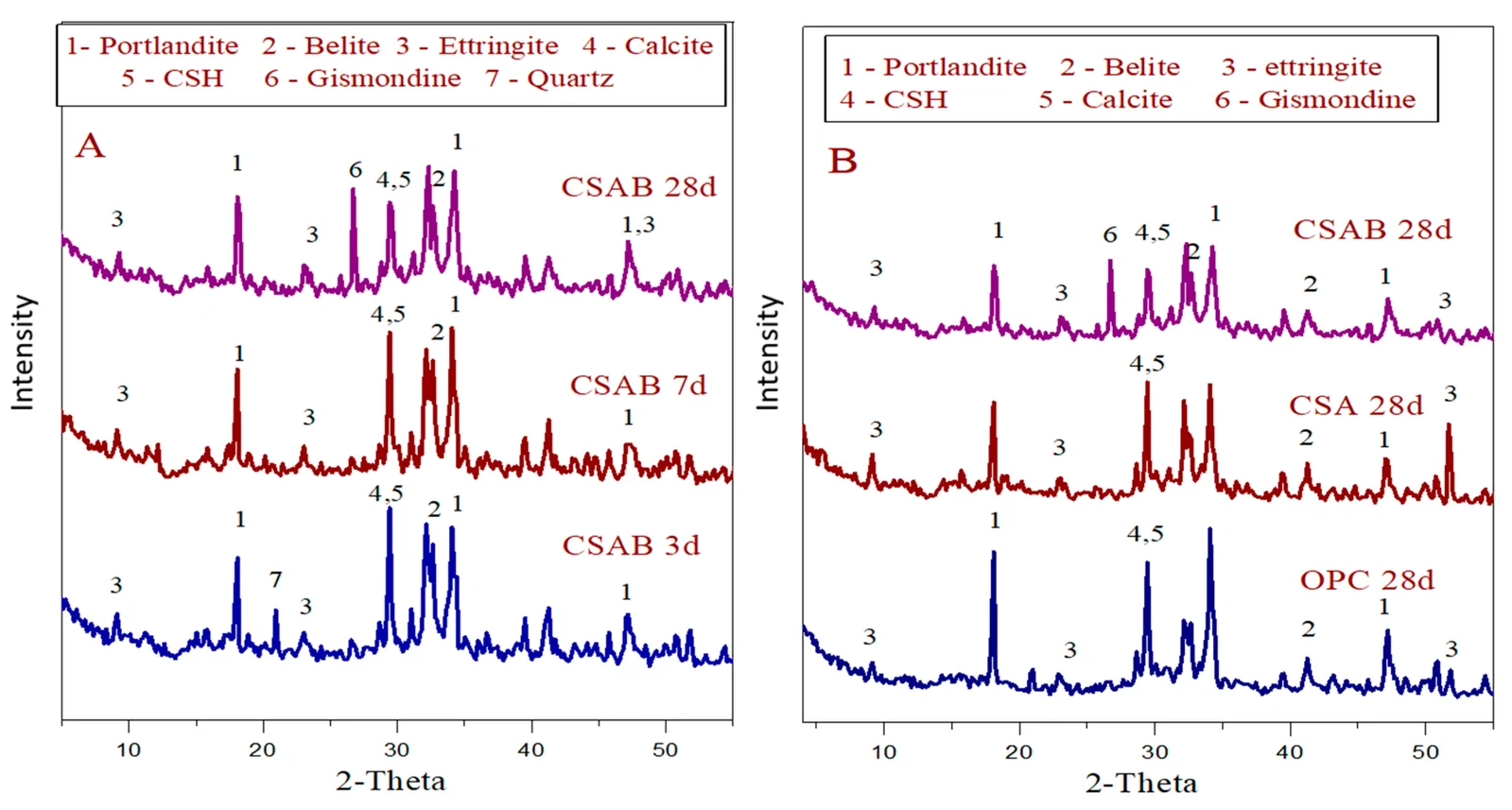
XRD patterns of (**A**) the CSAB cement past treated for 3, 7, and 28 days; the (**B**) OPC, CSA, and CSAB cements treated for 28 days.

The XRD analysis of CSAB during curing time for up to 28 days is shown in Figure 2A. The presence of ettringite, portlandite, alite, C_2_S, calcite, and CSH, along with C_4_Al_8_Si_8_O_32_∙16H (gismondine), is displayed in the figure. The results indicated that the intensity of portlandite and calcium silicate hydrate increased, while that of β-C_2_S decreased during the curing time up to seven days despite the continuous β-C_2_S phase hydration and the cement clinker producing CSH and portlandite. Upon prolonged hydration, Ca(OH)_2_ gradually reduced up to 28 days [32]. Meanwhile a new peak of gismondine could be observed [33]. This could be ascribed to the fact that during the exhaustion of CaSO_4_, the highly reactive added β-C_2_S or CSH as a source of silica was able to react with the formless Al(OH)_3_ and Ca(OH)_2_, thus forming gismondine. This result was parallel with the highly decrease in β-C_2_S and CSH contents at 28 days of hydration.

Figure 2B shows the XRD pattern of ordinary, CSA, and CSAB cements that were hydrated for 28 days. The results showed that the portlandite and CSH decreased with the substitution of 10% OPC with an equal amount of calcium sulfoaluminate phase. Additionally, the substitution of more 10 wt% β-C_2_S in addition of calcium sulfoaluminate tended to decrease the intensity of CSH, portlandite, and gismondine. This was due to the reaction of CSH or β-C_2_S with AH_3_. This AH_3_ was generated from the hydration of C_4_A_3_S, as shown in the equation:

C_4_A_3_S + H → C_3_A·3CS H_32_ + AH_3_ (3)

AH_3_ is an amorphous phase and could not be detected by the XRD. The ettringite, however, was detected and was found to increase in presence with the addition of 10% calcium sulfoaluminate; then, it decreased after the substitution of more 10% β-C_2_S.

**Figure 3:**
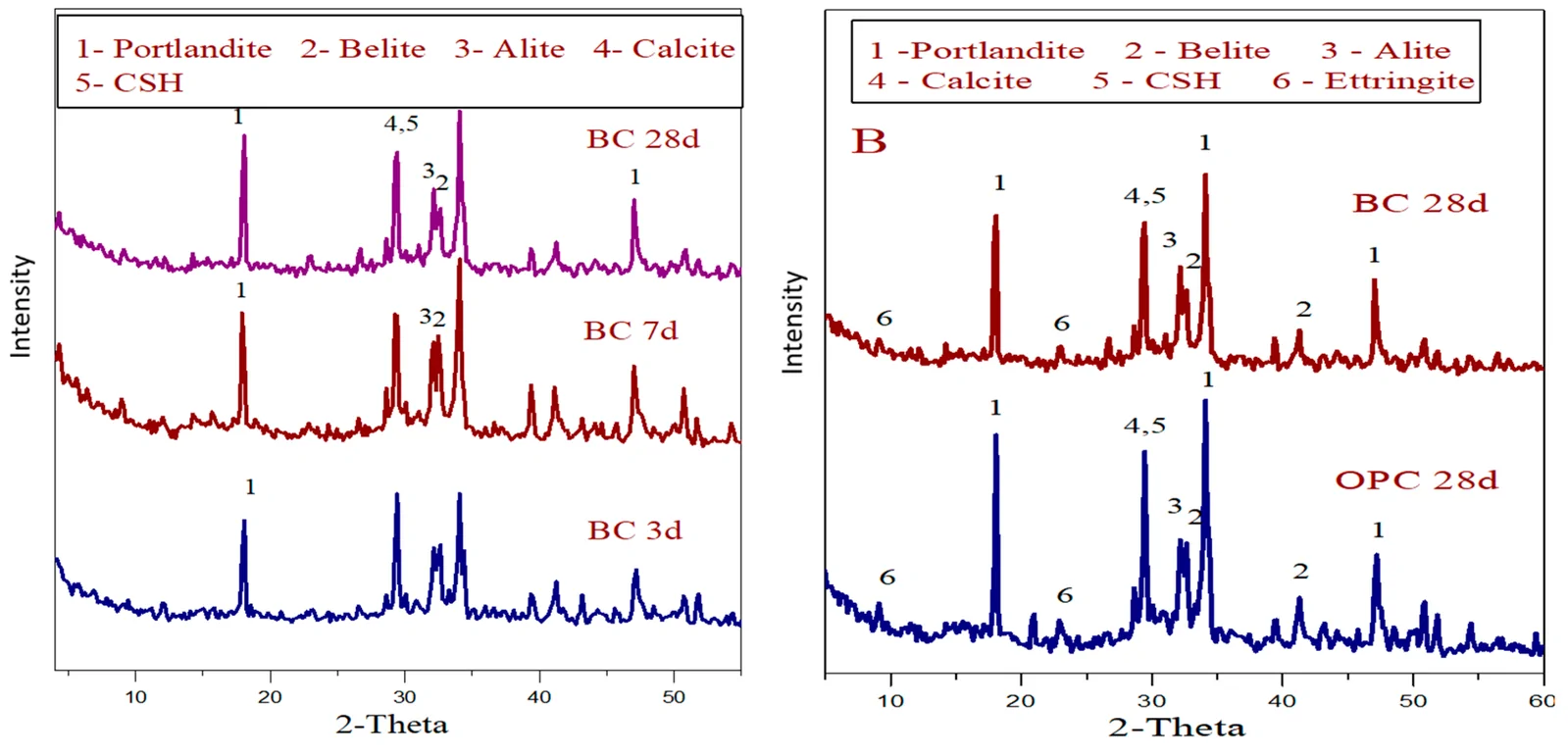
XRD patterns of (**A**) the BC cement paste cured for 3, 7, and 28 days; (**B**) OPC and BC cement pastes cured for 28 days.

The XRD patterns of the BC cement during the hardening period is illustrated in Figure 3A. Samples hydrated for three days showed the appearance of Ca(OH)_2_, CSH, β-C_2_S, alite, and calcite. The calcite overlapped by the CSH. The results indicate that the intensity of portlandite peak rises along with the hardening period for up to 28 days because of the hydration of cement clinkers like dicalcium and tricalcium silicate. It could be noticed that the intensity of β-C_2_S highly decreased, while that of CSH increase along with the curing time for up to 28 days; this was related to the unceasing hydration of the β-C_2_S phase. Figure 3B shows the XRD patterns of the OPC and BC cements during the hardening period. The results indicated that the intensity of the Ca(OH)_2_ peak was reduced by adding the β-C_2_S phase, that caused by the reduction of the cement clinker as C_3_S, which produced a high amount of portlandite. The increase of the calcite phase in the case of OPC was largely caused by the increase of the released portlandite, which could be easily carbonated.

#### 3.2.3. DSC Analysis

**Figure 4:**
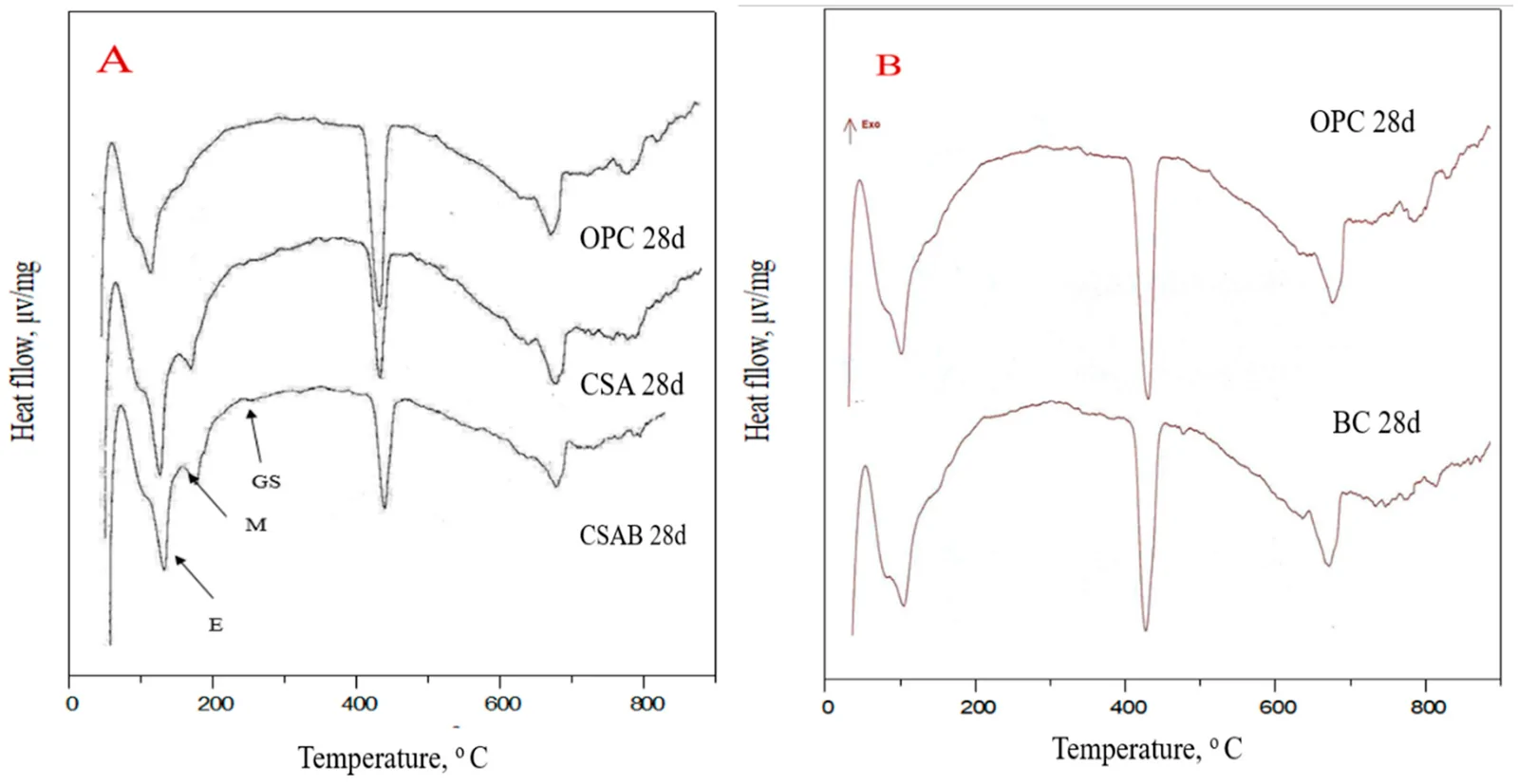
The DSC patterns of (**A**) OPC, CSA, and CSAB cement pastes; the (**B**) OPC and β-C_2_S BC cement pastes cured for 28 days.

The DSC patterns of the OPC, CSA, and CSAB cements as a function of the hardening period for up to 28 days are illustrated in Figure 4A. The thermogram for the OPC that was hydrated for 28 days shows endoergic peaks at 87, 112, 460, and 750 °C. The endoergic points under 200 °C were mainly caused by the dehydration of calcium sulfoaluminate hydrates as well as CSH (calcium silicate hydrate) The endoergic points at 460 and 750 °C were caused by the decay of calcium hydroxide and the decomposition of amorphous and crystalline CaCO_3_. The figure also shows an endoergic point of portlandite reductions after the addition of calcium sulfoaluminate. On the other hand, replacement OPC with more β-C_2_S tend to decrease the intensity of the portlandite. This may have been related to the portlandite’s reaction with CSA, which provided ettringite, or it may have been due to the reduction of cement clinkers, such as C_3_S and C_2_S, which produced more portlandite. In case of CSAB, the endoergic peak of portlandite was smaller than that of CSA. This was caused by the reaction of portlandite with CSH and AH_3_ that gave calcium sulfoaluminate hydrates. The thermograms of sulfoaluminate cement pastes hydrated for 28 days show a potent endoergic point due to ettringite’s dehydration at 120 °C. The endoergic point observed at 200 °C could be ascribed to a solid solution of ettringite and monosulfate hydrate. Additionally, the thermograms of CSAB show the presence of C-S-H, ettringite, monosulfate hydrate, and a small spike at 300 °C related to gismondine dehydration. We can conclude from these thermograms that the core hydrating products were ettringite, monosulfate hydrate, and gismondine.

The DSC thermograms of the OPC and BC cements at 28 days of curing time are shown in Figure 4B. The thermograms show that the peaks of CSH were broader and larger than that of OPC due to the hydration of the β-C_2_S phase that formed CSH and the acceleration effect of the cement clinkers that produced more hydration products. On the other hand, the pike at 460 °C can be ascribed to the CH decomposition, which slightly decreased with the addition of β-C_2_S as a result of the decrease of cement clinkers. Furthermore, the low hydration of β-C_2_S at early stages led to a decrease in portlandite.

#### 3.2.4. IR Spectra

**Figure 5:**
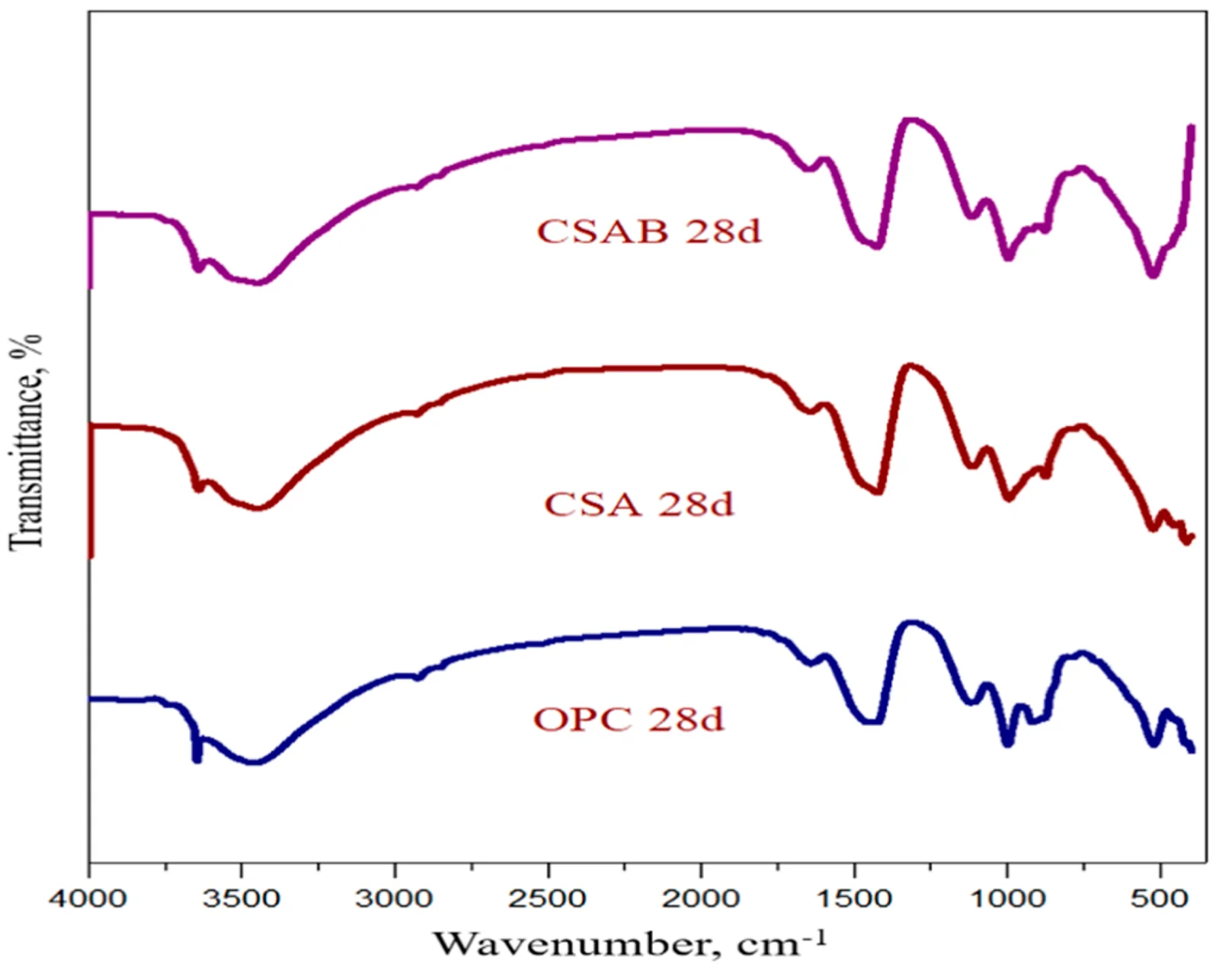
IR spectra of OPC, CSA, and CSAB cement pastes cured for 28 days.

The IR spectra of the OPC, CSA, and CSAB cement pastes cured for 28 days are illustrated in Figure 5. The figure shows that the band at 3439 cm^−1^ can be ascribed to the extending OH groups associated with H_2_O in hydrating products, namely C-S-H, ettringite, monosulfate hydrate, and CAH (calcium aluminate hydrate). Furthermore, the band at 1650 cm^−1^ is associated with H_2_O bending and specifies CSH development. The band at 876 cm^−1^ indicates the formation of an Al-O-H bond that shows prominence over the 976 cm^−1^ band pertaining to CSH, and this implies that the hydration of aluminates proceeded much faster than that of silicates. The band at 3647 cm^−1^ [34] signifies the presence of CaO produced from cement clinker hydration, namely C_3_S and C_2_S. The figure shows that the strength of the Ca(OH)_2_ band at 3645 cm^−1^ decreased with the addition of sulfoaluminate, with or without β-C_2_S, due to the reduction of C_3_S and C_2_S that generated a high content of portlandite during hydration. This decrease of portlandite was due to its reaction with CSH and AH_3_ that provided gismondine. The intensity of the band at 3439 cm^−1^ can be ascribed to extension of the OH-groups in the hydration products that increased with the addition of the sulfoaluminate phase due to the formation of ettringite, which has a high-water content. Meanwhile, the intensity of this band was slightly decreased with the addition of the sulfoaluminate-β-C_2_S mix to lower than that of the sulfoaluminate cement pastes. This was mostly caused by the reduction of cement clinkers, namely C_3_A and C_3_S, that contributed to the formation of hydration products. The intensity of the band at 972 cm^−1^ is due to the symmetric stretching of O-Si-O and O-Al-O [35] increase with CSAB cement pastes relied to the formation of Gismondine. The bands at 1439 and 873 cm^−1^ are because of carbonate.

#### 3.2.5. The Combined Water Contents

**Figure 6:**
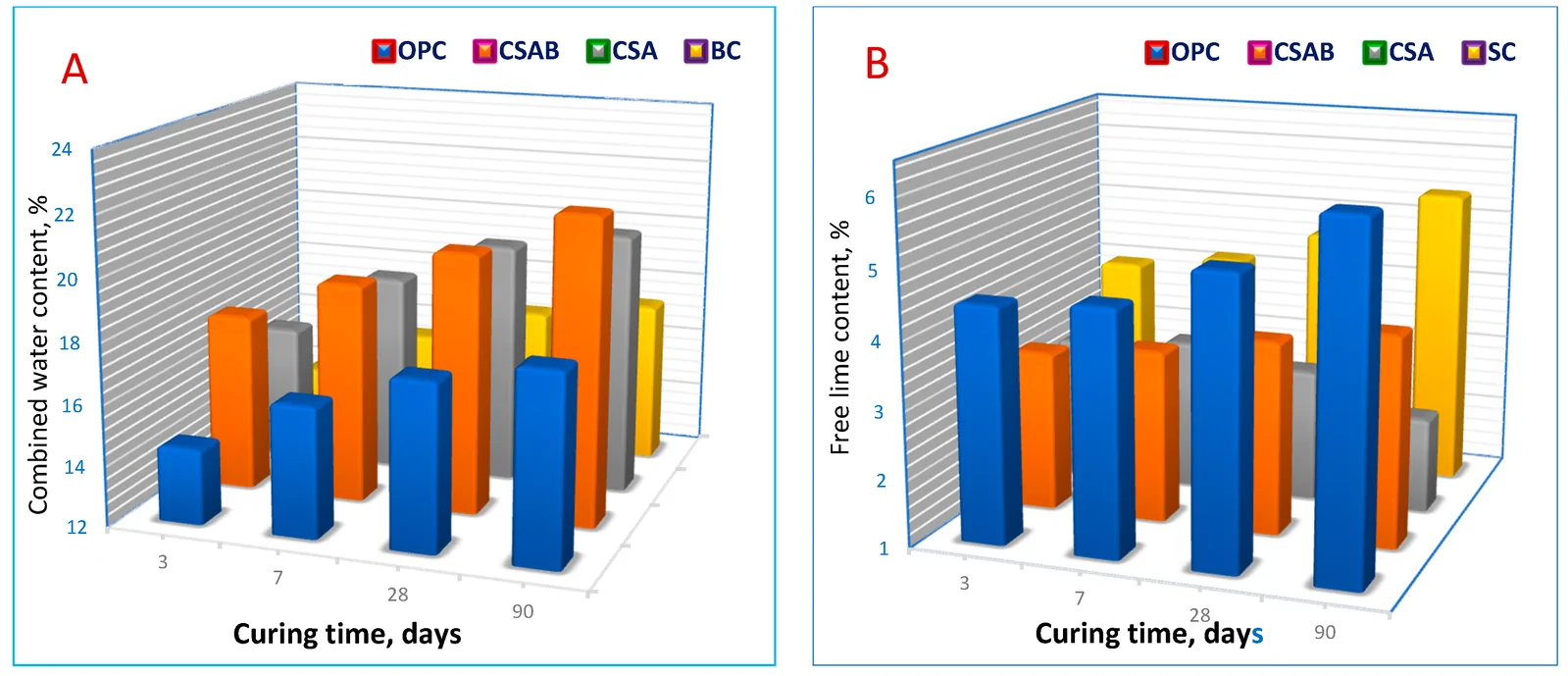
(**A**) The combined water content and (**B**) free lime content of the OPC, BC, CSA, and CSAB cement pastes cured for 3, 7, 28, and 90 days.

The combined water contents varied as a result of quantity and the kind of hydration product. The combined water contents generally increased during the hardening period because of the hydration progress and the formation of hydration products that contained great combined water contents, namely C-A-H, C_4_AH_13_, and C_2_ASH [36]. The chemically-combined water contents of the OPC, CSA, and CSAB cements during the hardening period are illustrated in Figure 6A. The chemically-combined water contents for the CSA and CSAB cements were greater than that of OPC [37] due to the quicker rate of reaction of the CSA mix forming sulfoaluminate hydrates with high water contents. The CSAB cements showed a greater and lower H_2_O content than OPC and CSA cements containing only 10% C_4_A_3_S, respectively. This was caused by the reduction of OPC and the presence of 10% β-C_2_S, which has a lower rate of hydration than OPC and calcium sulfoaluminate. Evidently, the addition of the β-C_2_S phase slightly decreased the combined water content, as compared to OPC, for up to 90 days. This was mostly due the reduction of OPC at the expense of β-C_2_S. Additionally, the addition of β-C_2_S have only one mole of portlandite, which also decreased the chemically-combined water contents of the β-C_2_S cement pastes.

#### 3.2.6. The Free Lime Contents

The free lime contents of the OPC, CSA, and CSAB cements during the hardening period are plotted in Figure 6B. The free CaO contents in the OPC and CSA cements increased during the hardening period for up to 90 days. This was due to the cement clinker hydration such as C_3_S and C_2_S, which formed calcium silicate hydrate and calcium hydroxide. Generally, CSA provided a lower CaO content than when only using OPC. This was due to the released lime from the OPC’s hydration reacting with CSA and providing ettringite, as well as AH_3_’s reaction with Ca(OH)_2_ that providing C-A-H. Therefore, the free lime content decreased for up to 90 days. The results also revealed that the free CaO content of the CASB cement increase during the hardening period for up to seven days. This was caused by the hydration of the portlandite generated from cement clinker and the hydration of β-C_2_S. Since the hardening period went on for about 90 days, the free CaO content decreased. These changes were caused by the consumption of portlandite during the development of gismondine. The results showed that β-C_2_S cements had a lesser CaO contents than those of the reference pastes. As previously discussed, this can be ascribed to the low β-C_2_S hydration rate and the decrease in the amount of cement clinkers, such as C_3_S, that produced more portlandite.

#### 3.2.7. The Compressive Strength

**Figure 7:**
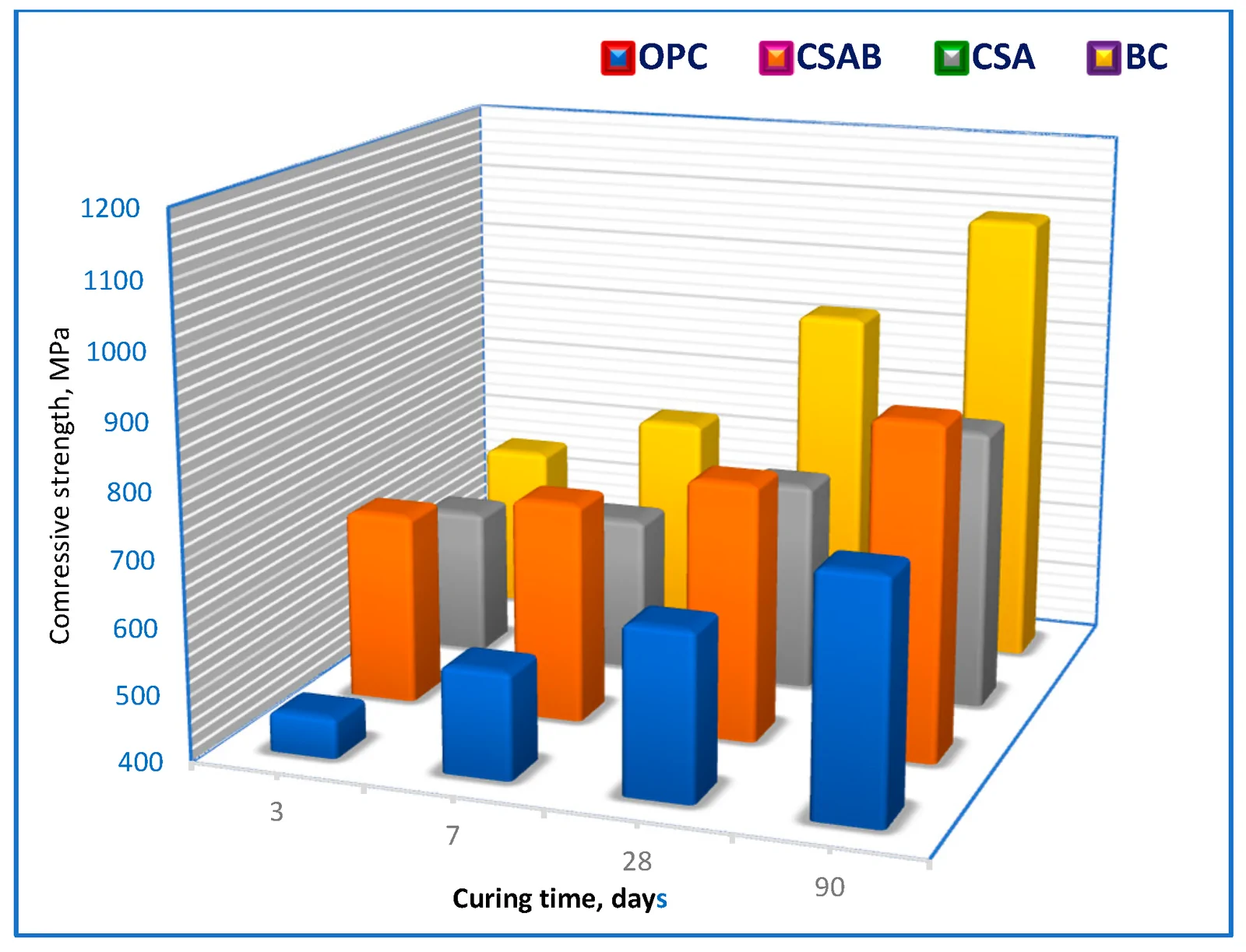
The compressive strength of OPC, BC, CSA, and CSAB cements during the hardening period.

The compressive strength of the OPC, CSA and CSAB cements during the hardening period is clearly plotted in Figure 7. The results illustrated that the compressive strength increase during the hardening period for every hydrated cement. This can be attributed to hydration progress and cementing material accumulation within the available space, thus giving higher strength. The data also showed that CSA cement pastes showed a higher early strength than OPC, mainly because of the quick ettringite formation in initial times that sealed some of the openings and densified the cement structure, subsequently raising compressive strength. The results showed that cements with 10% C_4_A_3_S had a more advanced strength than those containing 10% β-C_2_S and 10% C_4_A_3_S. The sulfoaluminate hydrate could link with the CSH gel and decrease the porosity, therefore increasing the strength. This was because of the major decrease in OPC, as well as the reduction in β-C_2_S’s hydration rate. Moreover, this increase may have also been due to the nano-CSA, which has high rate of hydration. The CASB cement pastes showed a more advanced strength in the initial and later times compared to that of the OPC cement pastes. This was mostly because the NPs in the β-C_2_S mix filled gaps between cement granules and accelerated the hydration of cement clinkers to form an extra dense structure; therefore, the compressive strength increase during the initial times. Later, the compressive strength increase because of β-C_2_S’s higher hydration at later ages and the formation of (Gismondine) contributes in the late strength [38].

Despite the β-C_2_S’s lower hydration rate during the initial times, it provided higher early and late strengths than those of OPC. This was basically caused by the reduction in water consistency and the acceleration effect of hydration, along with the 5% nano-β-C_2_S addition. Moreover, the deposition of nanoparticles in the spaces between cement grains densified the cement’s structure, and, accordingly, the compressive strength increase. Generally, the compressive strength of the mortar increased as the size of the particle decreased while a smaller particle size rendered a larger specific surface area, a larger reaction area [39].

### 3.3. Performance of OPC, CMS, and CMSB Cements

#### 3.3.1. The Water Consistency

The initial and final setting times and water consistency of the OPC, CMS and CMSB cements are shown in Table 2. The results showed the water consistency increase with the level of OPC-substitution by CMS or the CMSB phase. This was mainly due to the monosulfate phase’s hydration, which produced ettringite; that has high combined H_2_O content. Meanwhile, the monosulfate phase contained a high content of calcium sulfate and free lime; therefore, the mixing water content increased. Moreover, the upsurge in the surface area of the added resources tended to raise the water consistency. It was found that water consistency decreased when substituting 10% OPC by β-C_2_S. This was due to that the β-C_2_S phase had a lower hydration rate than OPC. Additionally, the initial and final setting times decreased with monosulfate mix/monosulfate and β-C_2_S addition compared to those of OPC, which was related to the very rapid hydration of the monosulfate mix that formed ettringite, which is known for fast hardening. Furthermore, the nanoparticles of the added materials acted as filler and accelerated the cement’s hydration, this creating more hydrating products that precipitated inside the holes and formed more dense structures. The initial and final setting times shortened with CMSB cement pastes due to water consistency reduction.

#### 3.3.2. X-ray Analysis

**Figure 8:**
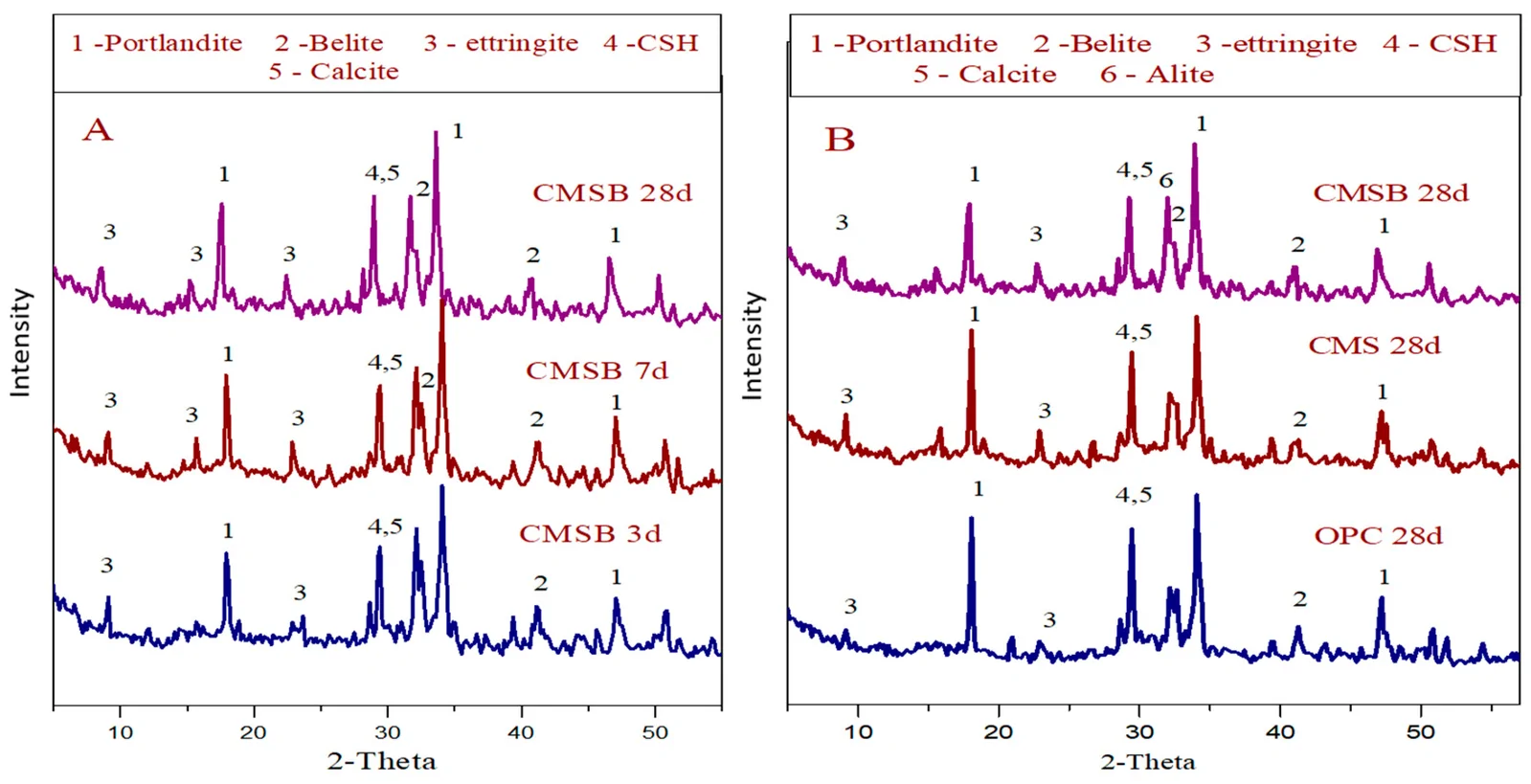
The XRD patterns of the (**A**) CMSB cement paste cured for 3, 7, and 28 days; the (**B**) CMSB, OPC, and calcium monosulfate cement pastes cured for 28 days.

The XRD patterns of the CMSB cements during the hardening period are shown in Figure 8A. The outcomes indicated that the intensity of ettringite was nearly the same from 3 to 28 days. This was caused by the complete hydration of C_4_A_3_S after 3 days. It should be noted that the strength of the β-C_2_S spike slightly decreased at 7 days due to the slower hydration rate of β-C_2_S during earlier times. This was sustained with the slight increase of CSH and portlandite until 7 days, that produced from the cement clinker hydration, and the added β-C_2_S that hydrated to form portlandite and CSH. As curing proceeded, the peaks of CSH with Ca(OH)_2_ increased with time due to the progress of hydration. Figure 8B shows the XRD patterns of the OPC, CMS and CMSB cements treated for up to 28 days. The figure shows that the strength of ettringite spike increased compared with the reference OPC after the addition of monosulfate. This was due to the hydration of monosulfate, which produced ettringite. In contrast, the addition of 10% monosulfate and 10% β-C_2_S to the OPC decreased the quantity of ettringite. The quantity of portlandite was lesser in the CMS and CMSB cement pastes than that of the OPC paste. This was caused by the substitution of 10% OPC by the β-C_2_S phase, which tended to decrease the content of the liberated portlandite. By comparing the XRD patterns of CMS with that of CMSB, we could see that the amount of liberated portlandite increase during the hardening period for up to 28 days. This was consistent with the trends of CSH lines that emerged in every hydrated cement, all of which decreased with the addition of monosulfate mix and β-C_2_S, related to the reduction of cement clinkers such as dicalcium silicate and tricalcium silicate.

#### 3.3.3. DSC Analysis

**Figure 9:**
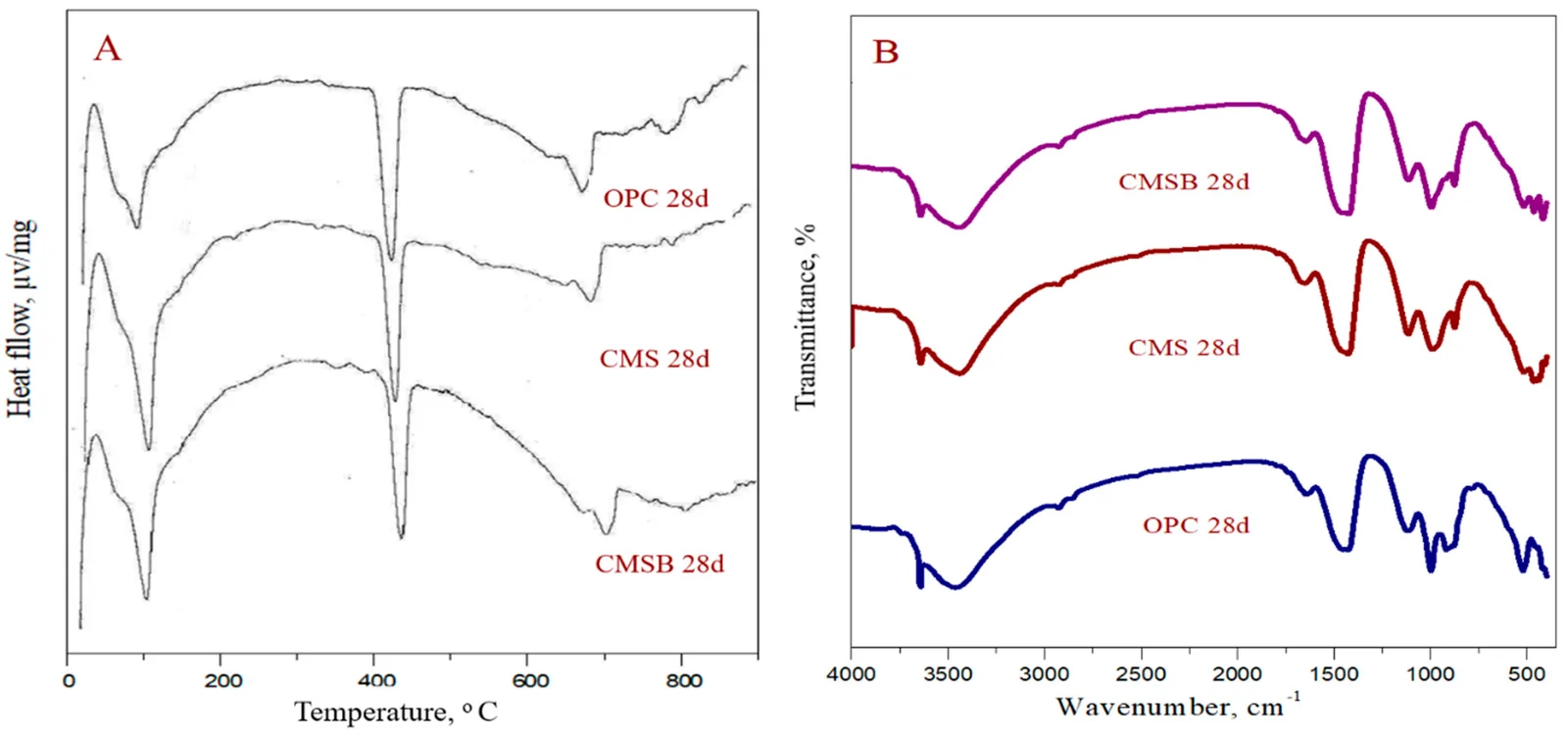
The DSC thermograms of the (**A**) OPC, CMS, and CMSB cement pastes; (**B**) IR spectra of the OPC, CMS, and CMSB cement pastes cured for 28 days.

The DSC thermograms of the hydrated OPC, CMS and CMSB cements treated for 28 days are shown in Figure 9A. The thermograms show the occurrence of few endoergic pikes at 60, 100, 460, and 770 °C. The endoergic pikes located at 60 and 100 °C were mainly caused by CSH dehydration and sulfoaluminate hydrates. Spikes at 460 and 770 °C represent the decomposition of portlandite and crystalline calcium carbonate, respectively. It is certain that the intensity of the sulfoaluminate hydrates was increased in the CMS cement pastes and was decreased in the CMSB cements due to OPC reduction. On the other hand, the intensity of Ca(OH)_2_ and CaCO_3_ was decreased in CMS and CMSB pastes due to the reduction of the cement clinkers. The DSC results are in a good agreement with the XRD conclusions.

#### 3.3.4. IR Spectra

Figure 9B shows the IR spectra of the OPC, CMS and CMSB cements treated for up to 28 days. The results showed that the band strength at 3496, 972, and 872 cm^−1^ of H_2_O, CSH, and CAH, respectively, increased with the addition of CMS to the cement pastes, relate to the continuous hydration of monosulfate and the cement clinkers producing more hydrating products. In contrast, the intensity of these peaks was slightly decreased in the CMSB cement pastes in comparison with that of the CMS cements. This was caused by OPC reduction. The intensity of the 3645 cm^−1^ band was caused by the decrease of portlandite in the CMS and CMSB cements. This was mostly because of the reduction of cement clinker such as C_3_S that formed more free lime. The band absorption at 1434 cm^−1^ and 875 cm^−1^ were formed by portlandite carbonation. These results are in good agreement with the XRD and DSC analyses.

#### 3.3.5. The Combined Water Contents

**Figure 10:**
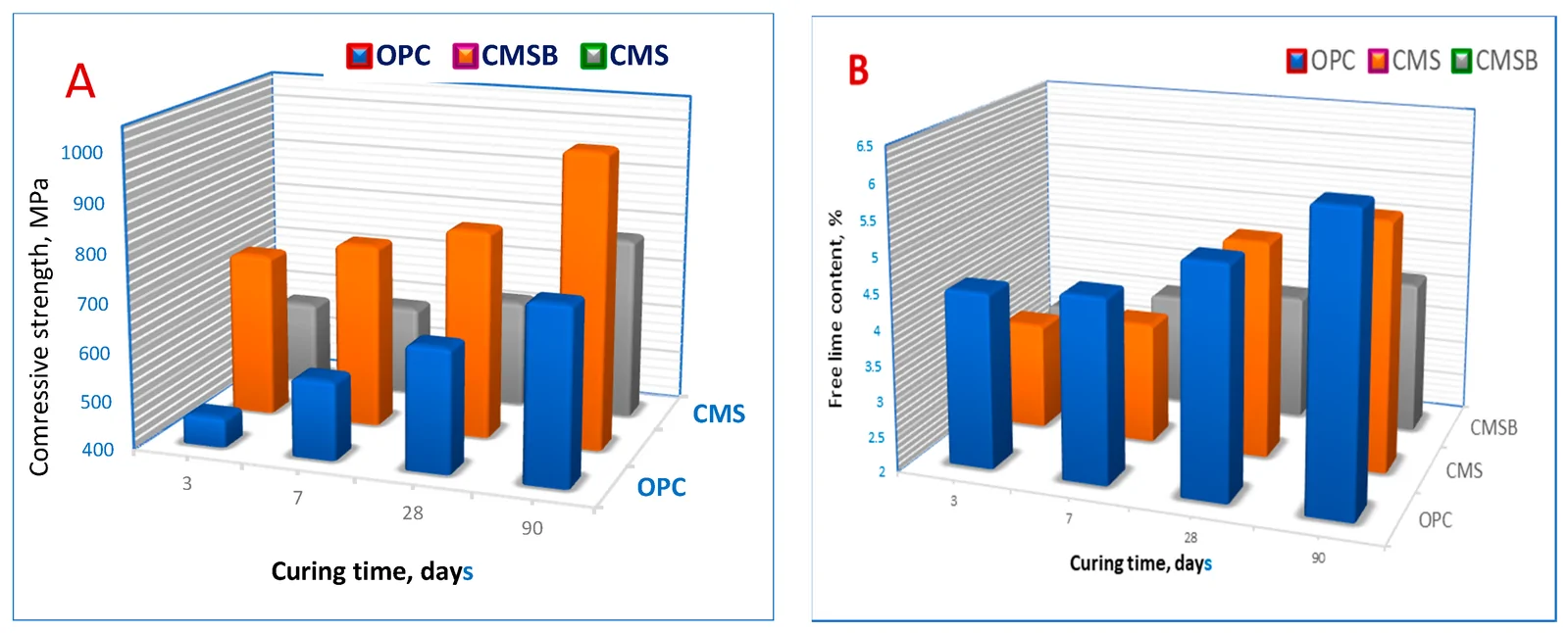
(**A**) The combined water content and (**B**) free CaO contents of the OPC, CMS, and CMSB cements during the hardening period.

The OPC, CMS, and CMSB cement pastes’ chemically-combined water contents during the hardening period are shown in Figure 10A. The results indicated that the chemically-combined water contents were higher in the CMS and CMSB cement pasts than those of plain OPC. This was due to the fact that the monosulfate mix had a high content of calcium sulfate; therefore, the probability of the formation of ettringite was higher, and ettringite formation increased with the increasing anhydrite level [33]. However, the ultra-fine elements of these phases acted like accelerators for cement clinker hydration, thus leading hydration and combined water content increases. The CMSB cement pastes showed a lesser chemically-combined H_2_O contents than those of the CMS cements. This was caused by the reduction of OPC, as well as the presence of 10% β-C_2_S, which has lower rate of hydration than OPC and C_4_A_3_S.

#### 3.3.6. The Free CaO Content

The OPC, CMS, and CMSB cements’ free CaO contents during the curing time are shown in Figure 10B. Although the monosulfate phase contained some amount of free lime, the results revealed that the CMS and CMSB cement pastes had lesser CaO contents than that of the OPC. This was due to the consumption of CaO during ettringite formation at early ages before three days. Moreover, adding CMSB phases to the OPC, and the low rate of hydration of β-C_2_S tended to a decrease in CaO contents.

#### 3.3.7. The Compressive Strength

**Figure 11:**
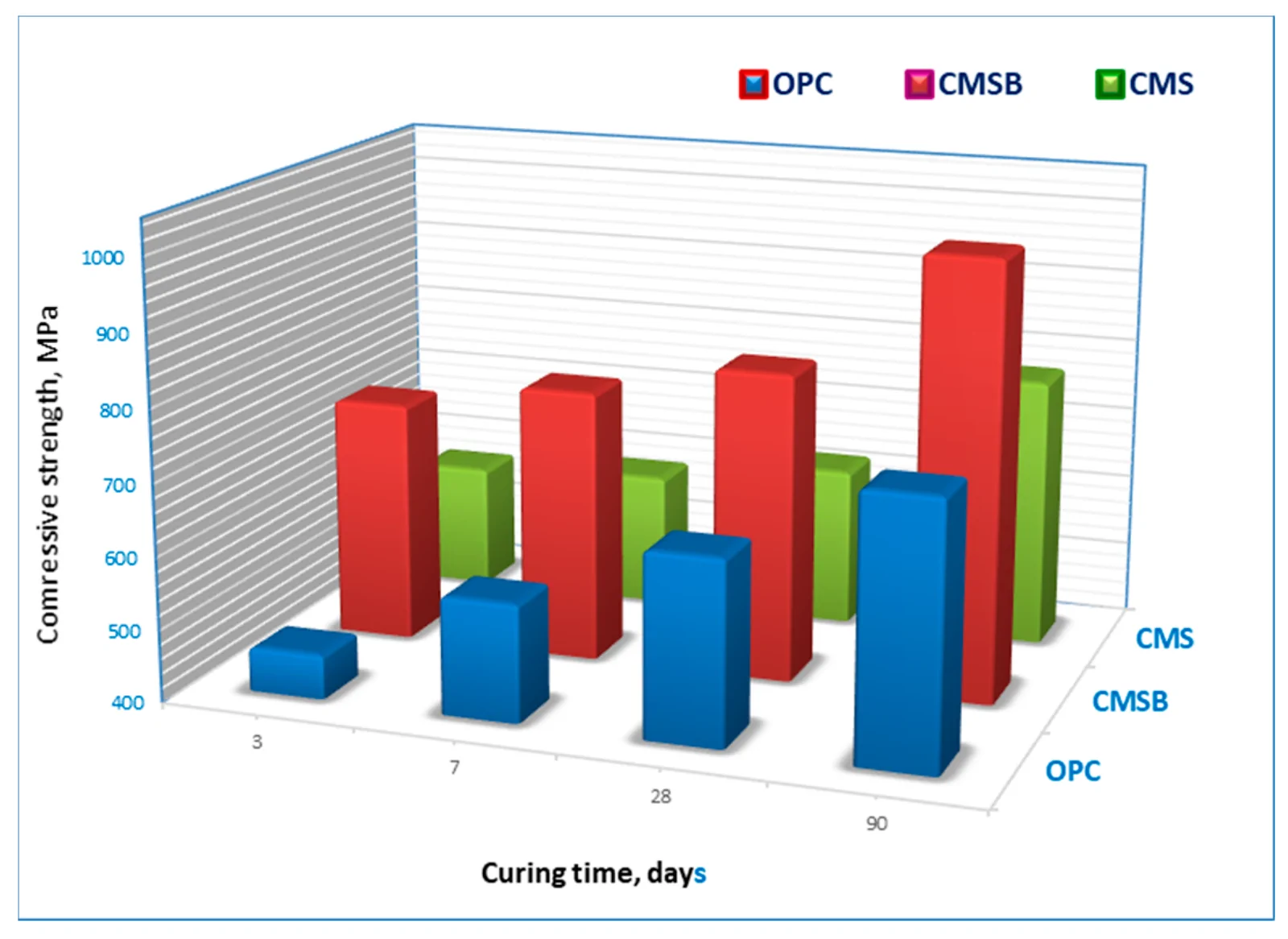
The compressive strength of the OPC, CMS and CMSB cements during the hardening period.

The OPC, CMS, and CMSB cements’ compressive strength during the hardening period are shown in Figure 11. The compressive strength increase with the hardening period for every cement as a result of the hydration of cementitious materials such as cement clinkers, as well as the formation of ettringite caused by C_4_A_3_S, CSH, and β-C_2_S hydration. The hydrating products precipitated inside holes, creating extra solid forms; as a result, the compressive strength increased. It was observed that C_4_AS addition to the OPC led to a higher early strength than the OPC by itself. These were associated with the involvement of ettringite formation. The results showed that the cements with C_4_AS had a more advanced strength than those containing C_4_A_3_S in addition to β-C_2_S. This was due to the fact that the sulfoaluminate hydrate could link with the CSH gel and decrease the porosity. Therefore, the strength increased. Additionally, the nanoparticles of the added phases had high rates of hydration and filled the pores, thus forming extra solid shapes; consequently, the compressive strength increased. On the other hand, the decrease in the compressive strength of the CMSB cement pastes was mostly because of the reduction in OPC clinkers and the slow rate of hydration of β-C_2_S.

On the other hand, the CMSB cement pastes showed a higher compressive strength than that of OPC at three days of hydration. The strength was decreased at 28 days and then increased at 90 days of hydration. This was demonstrates by the fact that when C_4_A_3_S was mixed with β-C_2_S and OPC, the formation of ettringite occurred under a high lime concentration caused by the hydration of the free CaO present in the clinker, the β-C_2_S hydration, or the OPC hydration—all of which led to some expansion inside the samples [40], consequently, the compressive strength decreased. The high compressive strength at later times was caused by β-C_2_S hydration.

## 4. Conclusions

Nanotechnology is an effective method to increase the endurance function of cement-based materials. This study researched the impact of adding nanomaterials, namely C_4_A_3_S, C_4_AS, and β-C_2_S, on green cement manufacturing with high-performance properties. The replacement of OPC by the nanophases greatly decreased the setting time and hastened cement hydration. The mechanical strength and endurance properties of the cements were significantly improved by using the nanophases. This was mostly due to the fact that the nanophases had large specific surface areas and also acted as ultra-fine aggregates that filled tiny voids in the cement, quickened the hydration process, and produced huge amounts of hydration products—thus increasing the mechanical strength. A few of the main discoveries from the research are as follows:

- We developed a new generation of high-performance concrete material with respect to their mechanical and endurance strength for sustainable construction.
- We reduced the construction expense and energy utilization, improved the bulk property, and increased the compressive strength of OPCs.
- We produced new concrete supplies using nanotechnology-based novel cement processing and cement pastes.
- We used waste materials and cementitious resources to validate sustainable environment, development, and economic impacts that are desired by countries that prioritize the significance of nanotechnology.

## Figures and Tables

**Table 1:** Chemical constitution of starting components (wt.%) and phase constitution of ordinary Portland cement (OPC).

Component	SiO_2_	Al_2_O_3_	Fe_2_O_3_	CaO	MgO	SO_3_	Na_2_O	I.L.
OPC	20.35	4.70	3.63	60.82	1.64	3.60	0.42	4.01
C_4_A_3_S	2.73	34.27	1.18	47.95	0.20	11.50	0.22	1.50
C_4_AS	1.10	17.24	0.66	70.52	0.33	17.55	0.31	4.07
β-C_2_S	30.82	0.45	0.07	65.71	0.32	0.02	0.68	1.92

**Table 2:** Composition of the prepared cements in wt%. CSA: calcium sulfoaluminate; CSAB: calcium sulfoaluminate-β-C_2_S; BC: β-C_2_S; CMS: calcium aluminum monosulfate; and CMSB: calcium aluminum monosulfate-β-C_2_S.

Group No.	Sample	OPC	C_4_A_3_S	(C_4_AS)	β-C_2_S	Water Consistency, %	Setting Time	
							Initial	Final
I	Mix (OPC)	100	00	00	00	26.6	120	295
	Mix (CSA)	90	10	00	00	27.0	45	250
	Mix (CSAB)	80	10	00	10	26.5	40	245
	Mix (BC)	95	00	00	5	25.0	95	285
II	Mix (OPC)	100	00	00	00	26.6	120	295
	Mix (CMS)	90	10	00	00	28.5	80	265
	Mix (CMSB)	80	10	10	10	27.3	65	255

## References

[1] Gupta V., Miura N. (2006). Influence of the microstructure on the super capacitive behavior of polyaniline/single-wall carbon nano-tube composites. J. Power Sources.

[2] Hanehara S., Ichikawa M. (2001). Nanotechnology of cement and concrete. J. Taiheiyo Cem. Corp..

[3] Daniyal M., Azam A., Akhtar S., Khan Z. (2018). Application of nanomaterials in civil engineering. Nanomaterials and Their Applications. Advanced Structured Materials.

[4] Coppola L., Coffetti D., Crotti E., Gazzaniga G., Pastore T. (2019). An empathetic added sustainability index (EASI) for cementitious based construction materials. J. Clean. Prod..

[5] Pelletier-Chaignat L., Winnefeld F., Lothenbach B., Müller C.J. (2012). Beneficial use of limestone filler with calcium sulphoaluminate cement. Constr. Build. Mater..

[6] Li J., Zhou C.Y., Yang Y.J. (2012). Optimization of mineral composition of high β -C2S-sulphoaluminate clinker. J. Chin. Ceram. Soc..

[7] Senff L., Castela A., Hajjaji W., Hotza D., Labrincha J.A. (2011). Formulations of sulfoβ-C2S cement through design of experiments. Constr. Build. Mater..

[8] Hewlett P.C., Liska M. (2004). Lea’s Chemistry of Cement and Concrete.

[9] Martín-Sedeño M.C., Cuberos A.J.M., de La Torre A.G., Álvarez-Pinazo G., Ordónez L.M., Gateshki M., Aranda M.A.G. (2010). Aluminum-rich β-C2S sulfoaluminate cements: Clinkering and early age hydration. Cem. Concr. Res..

[10] Zivica V. (2001). Possibility of the modification of the properties of sulfoaluminate β-C2S cement by its blending. Ceram. Silikáty.

[11] Glasser F.P., Zhang L. (2001). High-performance cement matrices based on calcium sulfoaluminate–β-C2S compositions. Cem. Concr. Res..

[12] Janotka I., Ray A., Mojumdar S.C. (2003). The hydration phase and pore structure formation in the blends of sulfoaluminate-β-C2S cement with Portland cement. Cem. Concr. Res..

[13] Quillin K. (2001). Performance of β-C2S–sulfoaluminate cements. Cem. Concr. Res..

[14] Liao Y., Wei X., Li G. (2011). Early hydration of calcium sulfoaluminate cement through electrical resistivity measurement and microstructure investigations. Constr. Build. Mater..

[15] Pera J., Ambroise J. (2004). New applications of calcium sulfoaluminate cement. Cem. Concr. Res..

[16] Gastaldi D., Canonico F., Boccaleri E. (2009). Ettringite and calcium sulfoaluminate cement: Investigation of water content by near-infrared spectroscopy. J. Mater. Sci..

[17] Heikal M., El-Didamony H., El-Sokkary T.M., Khalil K.A., Ahmed I.A. (2014). Active β-C2S and the hydration of calcium sulfoaluminates prepared from nano-materials. Ceram. Silikáty.

[18] El-Didamony H., Khalil K.A., Ahmed I.A., Heikal M. (2012). Preparation of b-dicalcium silicate (b-C2S) and calcium sulfoaluminate C3A3CS phases using non-traditional nano-materials. Constr. Build. Mater..

[19] Mathew L., Narayanankutty S.K. Synthesis and characterization of nanosilica. Proceedings of the International Conference on Advances in Polymer Technology.

[20] Abd El-Raoof F. (2008). Assessment of Developed Refractory Composites for Slide Gates of Continuous Steel Casting Systems. Ph.D. Thesis.

[21] ASTM (2008). ASTM Designation C-191. Standard Test Method for Normal Consistency and Setting Of Hydraulic Cement.

[22] El-Didamony H. (1980). Application of differental thermogravimetry to the hydration of expnsive cement pastes. Thermochim. Acta.

[23] Ahmed I.A., Al-Radadi N.S., Hussein H.S., Ragab A.H. (2019). Environmentally Friendly Mesoporous Nanocomposite Prepared from Al-Dross Waste with Remarkable Adsorption Ability for Toxic Anionic Dye. J. Chem..

[24] Ettarh C., Galwey A. (1996). Kinetic and mechanistic study of the thermal decomposition of calcium nitrate. Thermochim. Acta.

[25] Kurdowski W., Duszak S., Trybalska B. (1997). B-C2S produced by means of low temperature synthesis. Cem. Concr. Res..

[26] Ramanathan S., Halee B., Suraneni P. (2020). Effect of calcium sulfoaluminate cement prehydration on hydration and strength gain of calcium sulfoaluminate cement-ordinary portland cement mixtures. Cem. Concr. Compos..

[27] Li W., Yu J., Ma S., Hu Y., Ge D., Shen X. (2018). The properties and hydration of Portland cement containing calcium sulfoaluminate cement. Ceram. Silikáty.

[28] Si-Jun K., Keun-Hyeok Y., Gyu-Don M. (2015). Hydration Characteristics of Low-Heat Cement Substituted by Fly Ash and Limestone Powder. Mate.

[29] Ginebra M.P., Driessens F., Planell J. (2004). Effect of the particle size on the micro and nanostructural features of a calcium phosphate cement: A kinetic analysis. Biomaterials.

[30] Yasong Z., Jianming G., ChuanbeiLiu X., Chen Z. (2020). The particle-size effect of waste clay brick powder on its pozzolanic activity and properties of blended cement. J. Clean. Prod..

[31] Winnefeld F., Lothenbach B. (2010). Hydration of calcium sulfoaluminate cements—Experimental findings and thermodynamic modeling. Cem. Concr. Res..

[32] Antiohos S., Papageorgiou A., Tsimas S. (2006). Activation of fly ash cementitious systems in the presence of quicklime. Part II: Nature of hydration products, porosity and microstructure development. Cem. Concr. Res..

[33] Alons S., Palomo A. (2001). Calorimetric study of alkaline activation of calcium hydroxide-metakaolin solid mixtures. Cem. Concr. Res..

[34] Palomo A., Glasser F.P. (1992). Chemically-bonded cementitious materials based on metakaolin. Br. Ceram. Trans. J..

[35] El-Diadamony H., Amer A.A., Sokkary T.M., El-Hoseny S. (2018). Hydration and characteristics of metakaolin pozzolanic cement pastes. HBRC J..

[36] El-Gamal S., Amin M., Ramadan M. (2017). Hydration characteristics and compressive strength of hardened cement pastes containing nano-metakaolin. HBRC J..

[37] Zhen G., Yan X., Zhou H., Chen H., Zhao T., Youcai Z. (2011). Effects of calcined aluminum salts on the advanced dewatering and solidification/stabilization of sewage sludge. J. Environ. Sci..

[38] Haruehansapong S., Pulngern T., Chucheepsakul S. (2014). Effect of the particle size of nanosilica on the compressive strength and the optimum replacement content of cement mortar containing nano-SiO2. Constr. Build. Mater..

[39] Wu K., Shi H., Guo X. (2011). Utilization of municipal solid waste incineration fly ash for sulfoaluminate cement clinker production. Waste Manag..

[40] El-Didamony H., Radwan A., Khattab I., El-Alfi E.A., Mohammed M.S. (2014). Charcteristics of sulphate resisting cement pastes containing different ratios of β-c2s phase. J. Eng. Technol. Res..

