# Correction: Ahmed, I.A.; S. Al-Radadi, N. Estimating the Impact of Nanophases on the Production of Green Cement with High Performance Properties. *Materials* 2020, *13*, 4197

**DOI:** 10.3390/ma19173609

**Published:** 2026-08-25

**Authors:** Inas A. Ahmed, Najlaa S. Al-Radadi

**Affiliations:** 1 Department of Chemistry, Faculty of Science, King Khalid University, Abha 62224, Saudi Arabia; 2 Department of Chemistry, Faculty of Science, Taibah University, Al-Madinah Al-Munawarah 14177, Saudi Arabia; nsa@taibahu.edu.sa

In the original publication [1], Figures 1 and 2 have been removed.

“Ahmed, I.A.; Al-Radadi, N.S.; Hussein, H.S.; Ragab, A.H. Environmentally Friendly Mesoporous Nanocomposite Prepared from Al-Dross Waste with Remarkable Adsorption Ability for Toxic Anionic Dye. *J. Chem.* **2019**, *2019*, 7685204” was not cited. The citation has now been inserted into Section 3.1. Characteristics of Nanophases, Paragraph 1 and should read:

Chemical constitution of starting components (wt.%) and phase composition of ordinary Portland cement (OPC) are shown in Table 1. The XRD and TEM characterization of the synthesized nano-silica and aluminum hydroxide was previously reported in our earlier study [23]. The XRD patterns confirmed that the obtained nano-SiO_2_ was completely amorphous, while aluminum hydroxide consisted mainly of gibbsite and bayerite phases with a low degree of crystallinity. The TEM analysis revealed that the average particle sizes of nano-silica and aluminum hydroxide were approximately 13 nm and 38 nm, respectively.

Following this correction, the order of some references has been adjusted accordingly.

In Section 3.1, Paragraph 2, the sentence “The XRD-patterns of the β-C_2_S, calcium sulfoaluminate, and calcium aluminum monosulfate nanophases are shown in Figure 1B.” is updated to “The XRD-patterns of the β-C_2_S, calcium sulfoaluminate, and calcium aluminum monosulfate nanophases are shown in Figure 1.”.

The authors state that the scientific conclusions are unaffected. This correction was approved by the Academic Editor. The original publication has also been updated.
